# The pathogenic role of retinoid nuclear receptor signaling in cancer and metabolic syndromes

**DOI:** 10.1084/jem.20240519

**Published:** 2024-08-12

**Authors:** Mark Esposito, John K. Amory, Yibin Kang

**Affiliations:** 1 Department of Molecular Biology, Princeton University, Princeton, NJ, USA; 2 Kayothera, Inc, Seattle, WA, USA; 3 University of Washington, Seattle, WA, USA; 4 Ludwig Institute for Cancer Research Princeton Branch, Princeton, NJ, USA

## Abstract

The retinoid nuclear receptor pathway, activated by the vitamin A metabolite retinoic acid, has been extensively investigated for over a century. This study has resulted in conflicting hypotheses about how the pathway regulates health and how it should be pharmaceutically manipulated. These disagreements arise from a fundamental contradiction: retinoid agonists offer clear benefits to select patients with rare bone growth disorders, acute promyelocytic leukemia, and some dermatologic diseases, yet therapeutic retinoid pathway activation frequently causes more harm than good, both through acute metabolic dysregulation and a delayed cancer-promoting effect. In this review, we discuss controlled clinical, mechanistic, and genetic data to suggest several disease settings where inhibition of the retinoid pathway may be a compelling therapeutic strategy, such as solid cancers or metabolic syndromes, and also caution against continued testing of retinoid agonists in cancer patients. Considerable evidence suggests a central role for retinoid regulation of immunity and metabolism, with therapeutic opportunities to antagonize retinoid signaling proposed in cancer, diabetes, and obesity.

## Teaser

There are myriad conflicting hypotheses surrounding the retinoid nuclear receptor pathway. This review integrates key clinical, genetic, and pharmacologic data to suggest human disease settings where retinoic acid nuclear signaling plays a pathogenic role and suggests opportunities for therapeutic intervention.

## Introduction

**Figure 1.**
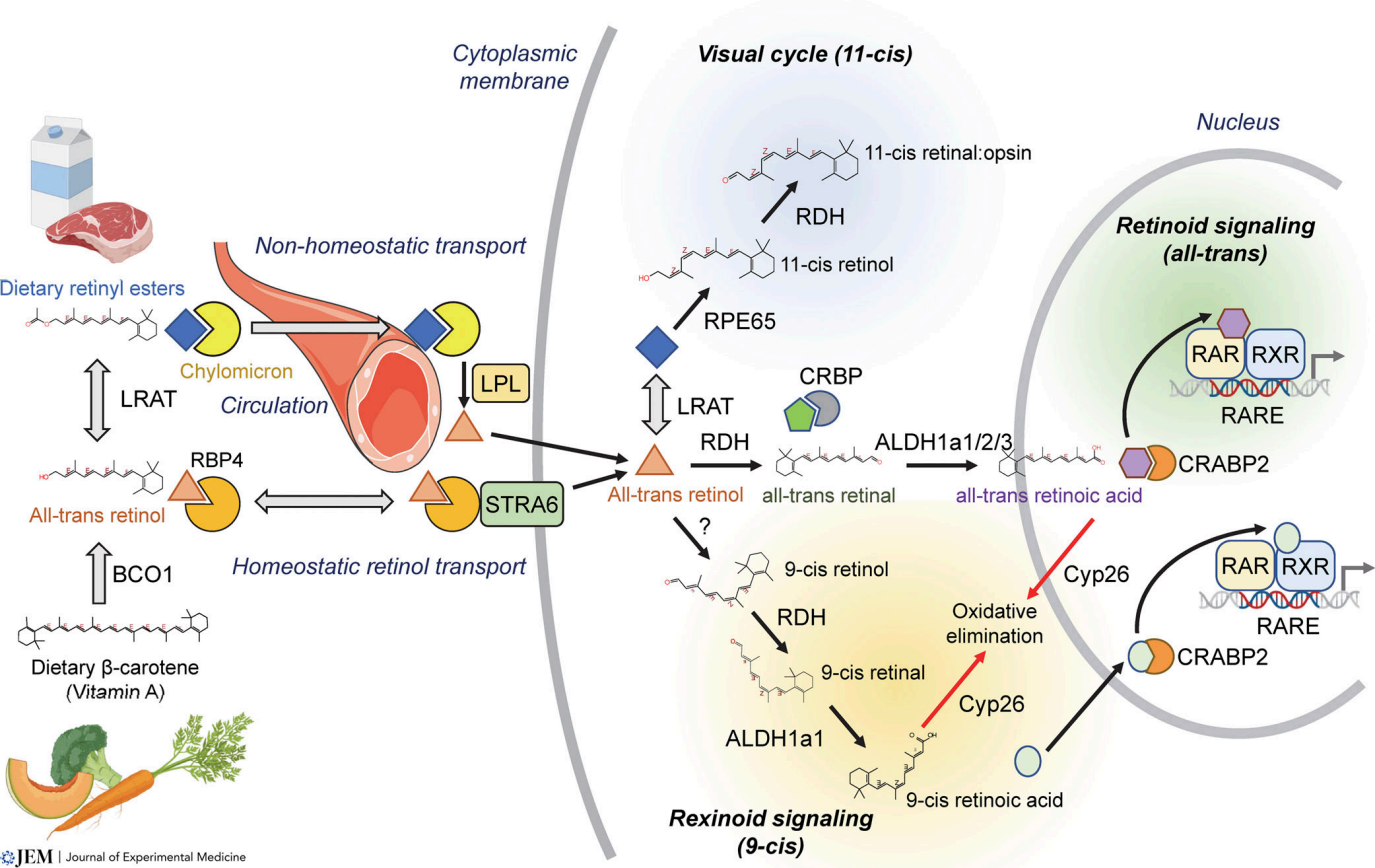
**Retinoid metabolism branches into nuclear signaling and non-nuclear signaling arms depending on stereoisomerization.** Retinoid metabolism is initiated by dietary intake of β-carotene (vitamin A), which is processed by β-carotene 15–15′-oxygenase (BCO1) through oxidative cleavage to retinol for transport, or further converted by LRAT to retinyl esters for storage in the liver. All-trans retinol is loaded onto retinol binding protein 4 (RBP4) in the liver and secreted to maintain plasma retinol homeostasis. Circulation and binding of RBP4 to the STRA6 transporter in target cells leads to the import of all-trans retinol. A subsidiary source of retinol is derived from retinyl esters that are transported through the bloodstream by chylomicrons in a non-homeostatic mechanism where they are released by LPL. In retinal cells, the LRAT-RPE65 stereoisomerization pathway generates 11-cis retinol that is oxidized to 11-cis retinal(dehyde) by retinol dehydrogenase (RDH) enzymes. Covalent binding to opsin and subsequent photostereoisomerization to all-trans retinal leads to synaptic transmission and vision. The rexinoid pathway is driven by stereoisomerization of all-trans retinol to 9-cis retinol through unknown mechanisms, RDH enzymes then oxidize 9-cis retinol to 9-cis retinal. 9-cis retinal is oxidized by aldehyde dehydrogenase 1a1 (ALDH1a1) to generate 9-cis retinoic acid, which is the ligand for rexinoid (RXR) signaling. The canonical branch of nuclear retinoid signaling is stimulated by conversion of CRBP-bound all-trans retinol to all-trans retinal by the RDH enzymes, which is then oxidized by tissue-specific expression of either ALDH1a1, ALDH1a2, or ALDH1a3 to generate the obligate RAR ligand, atRA. Both 9-cis and atRA are shuttled into the nucleus by cellular retinoic acid binding protein 2 (CRABP2) to activate nuclear signaling and are oxidized by CYP26 to deactivate nuclear receptor activity. Unknown factors govern the paracrine secretion of atRA. Illustration created in part with Biorender.com.

The retinoid pathway is complex. Conventional nuclear retinoid signaling is activated when all-trans retinoic acid (atRA), an oxidized metabolite of dietary vitamin A, binds the retinoic acid nuclear receptors (RARs). This binding results in changes in transcription, including morphogenic activity in development, regulation of immune tolerance and trafficking, spermatogenesis, as well as control of metabolic pathways (Altucci et al., 2007; Duester, 2008; Hall et al., 2011b). In addition to the conventional branch of retinoid signaling that activates the RAR nuclear receptors, the vitamin A pathway diverges into three other branches that are governed by stereoisomerization at the 9, 11, and 13 positions of retinol: the visual cycle in the eye enabled by 11-cis retinaldehyde, non-nuclear regulation by 13-cis retinoids, and rexinoid signaling activated by 9-cis retinoic acid (Fig. 1).

There is considerable complexity in the nuclear signaling pathway as the RAR/rexinoid X receptor (RXR) nuclear receptors participate in gene regulation in both the apo- and retinoic acid–bound states (Germain et al., 2002). These receptors are characterized by complex splicing regulation at the N-terminus that can result in context-dependent signaling, and both RAR and RXR receptors participate in non-retinoid nuclear receptor heterodimer signaling (Rochette-Egly and Germain, 2009). The combination of this biological complexity with unique technical hurdles, such as photoinstability of retinoid stereoisomers, low nanomolar concentrations in tissues, and lipophilicity of the retinoids, have all combined to make this pathway a difficult topic for study (Kane et al., 2005).

Despite this complexity, the retinoid pathway was one of the first discovered biological pathways; nutritional deprivation studies in the 1800s described effects later traced to retinol deficiency, and retinol was definitively isolated in 1918 as vitamin A (Semba, 2012). Since this discovery, the metabolic cascade from retinol intake to elimination has been well-described (Fig. 1). Whereas vitamin A is a precursor to four divergent pathways, synthetic retinoids that specifically activate each signaling branch were subsequently generated and have been used both in research and clinical treatment settings. Key advances from these efforts include approved RAR agonists for acute promyelocytic leukemia (tretinoin; synthetic atRA) and fibrodysplasia ossificans progressiva (palovarotene, RARγ agonist), 13-cis retinoic acid (isotretinoin/Accutane), and adapalene for dermatological applications to activate the non-nuclear pathway; bexarotene in cutaneous T cell leukemia (CTCL) to activate rexinoid signaling, and aciretin (mixed mechanism) for psoriasis and discoid lupus.

These rare disease approvals for synthetic RAR agonists were fortuitous; while the biological rationale is clear in retrospect, retinoids have been tested in hundreds of diseases over the last 50 years. This rapid proliferation of investigational testing was first enabled in the late 1970s by the relatively benign profile of 13-cis retinoic acid used in dermatology (Koeffler, 1983). Spanning to the present day, the body of evidence for retinoid agonists includes thousands of retinoid agonist trials conducted on hundreds of thousands of patients. While these trials have yielded ambivalent and often adverse outcomes (Table 1), retinoid agonists continue to be tested in the same diseases (Nagai and Ambinder, 2023).

**Table 1.** Controlled, randomized clinical trials or high-powered retrospective trials with high-quality analysis assessing the impact of a retinoid compared to a placebo or other appropriate control

Intent to treat	Drug	Mechanism	Design	Population	Cancer effects	Cardiometabolic effects	Trial ID	Sample size	CoMed	Note
**Prevention trials**										
	β-Carotene	Vitamin A	RCT	High-risk male smokers	**18% increase lung cancer**	**11% increase heart disease**	ATBC	29,133	Tocopherol	
	β-Carotene/retinyl palmitate	Vitamin A	RCT	High-risk smokers/asbestos	**28% increase lung cancer, 52% prostate cancer**	**26% increase cardiovascular mortality**	CARET	18,314	None	
	β-Carotene/retinol	Vitamin A	Retrospective	Healthy individuals (ages 50–76)	**53% increase lung cancer**	Not assessed	VITAL	77,126	Lutein	
	Fenretinide	RAR agonist	RCT	Healthy women	No overall effect	Not assessed	PMID: 10547391	2,972	None	^a^
	Fenretinide	RAR agonist	RCT	High-grade cervical squamous lesions	**40% decrease in response rate**	Not assessed	PMID: 11705848	39	None	
**Intervention trials**										
Melanoma	Retinol	Vitamin A	RCT	High-risk resected melanoma	No effect	Not assessed	PMID: 7931474	248	None	^b^
HNSCC	Retinyl palmitate	Vitamin A	RCT	HNSCC	Trend to twofold increase in recurrence	Not assessed	PMID: 9039219	106	None	
HNSCC	Retinyl palmitate	Vitamin A	RCT	Early HNSCC and lung cancer	No effect	Low-requency hypercholesterolemia and liver enzyme elevation	EUROSCAN	2,592	N-acetylcysteine	^c^
Lung cancer	Retinyl palmitate	Vitamin A	RCT	Stage 1 NSCLC	Trend to improved disease-free survival (P = 0.054)	**Hyperlipidemia and GGT elevation**	PMID: 8391063	307	None	
Lung cancer	Tamibarotene	RAR agonist	RCT	Stage IIIB/IV NSCLC	PFS HR 1.54 (P = 0.088). OS HR 1.48 (P = 0.24)	**Grade 3/4 hypertriglyceridemia**	NCT01337154	137	Paclitaxel/carboplatin	^d^
Lung cancer	atRA	RAR agonist	RCT	Advanced SCLC	No effect	No effect	NCT00617409	69	Chemo + DC vaccine	
Liver cancer	Amsilarotene	RARα agonist	RCT	Advanced HCC	No effect	**Deep vein thrombosis**	NCT00687596	52	None	
Cervical cancer	Alitretinoin	RAR/RXR agonist	RCT	CIN 2/3 cervical dysplasia	No effect	**Hyperlipidemia and decreased HDL**	PMID: 12582020	114	None	
Lung cancer	Bexarotene	RXR agonist	RCT	Advanced NSCLC	Trend to reduced OS/PFS	**Hyperlipidemia and hypothyroidism**	Spirit I	623	Cisplatin/vinorelbine	
Lung cancer	Bexarotene	RXR agonist	RCT	Advanced NSCLC	Trend to reduced OS/PFS (P = 0.06)	**Hyperlipidemia**	Spirit II	612	Carboplatin/paclitaxel	
Lung cancer	Isotretinoin	Non-nuclear retinoid	RCT	Stage 1 NSCLC	**56% increased death in smokers (P = 0.01)**	No significant differences	PMID: 11309437	1,166	None	
HNSCC	Isotretinoin	Non-nuclear retinoid	RCT	Stage 1/2 HNSCC	No effect	None	PMID: 16595780	1,190		
Renal cancer	Isotretinoin	Non-nuclear retinoid	RCT	Metastatic RCC	31% increased survival	None	EORTC 30951	320	Interferon α	
Neuroblastoma	Isotretinoin	Non-nuclear retinoid	RCT	Pediatric high-risk neuroblastoma	Trend to improved survival (P = 0.054)	None	CCG-3891	379		
Neuroblastoma	Isotretinoin	Non-nuclear retinoid	RCT	Pediatric stage 3/4 neuroblastoma	No effect	None	PMID: 11027423	175		
Glioblastoma	Isotretinoin	Non-nuclear retinoid	CT	Glioblastoma	Trend to worse outcomes	None	NCT00112502	178	TMZ, thalidomide, celecoxib	
Lung cancer	Disulfiram	ALDH1a1/2 inhibitor	RCT	Stage 4 NSCLC	**Improved PFS and OS**	Not assessed	PMID: 25777347	40	Cisplatin/vinorelbine	
**Topical gel trials**										
AIDS Kaposi sarcoma	Alitretinoin (topical)	RAR agonist	Within patient trial	Cutaneous Kaposi sarcoma	**16% increased response rate**	None	PMID: 11115156	115		
CTCL	Bexarotene (topical)	RXR agonist	Within patient trial	Early CTCL	**46% response rate**	None	PMID: 14576658	50		
Keratinocyte carcinoma	Tretinoin (topical)	RAR agonist	RCT	Men with history of KC	No overall effect	**Fourfold increase in death due to vascular disease**	VATTC	1,131	None	^e^
**Noncancer trials**										
Immune thrombocytopenia	atRA	RAR agonist	RCT	Steroid-resistant thrombocytopenia	**47% improved response**	None	PMID: 28917657	96	Danazol	

Key trial attributes along with cancer and cardiometabolic effects are listed. Bold indicates significant effects. Orange shading indicates adverse effect. Green shading indicates beneficial effect. HNSCC, head and neck squamous cell carcinoma; RCT, randomized controlled trial; CT, clinical trial; KC, keratinocyte carcinoma; NSCLC, non-small cell lung cancer; RCC, renal cell carcinoma; CIN, cervical intraepithelial neoplasia; HCC, hepatocellular carcinoma; SCLC, small cell lung cancer; PFS, progression-free survival; HR, hazard ratio; OS, overall survival; GGT, gamma-glutamyl transferase; VITAL, VITamin D and OmegA-3 TriaL; EUROSCAN, European Study on Chemoprevention with Antioxidants; EORTC, European Organisation for Research and Treatment of Cancer; CoMed, combination medication; TMZ, temozolomide.

a Beneficial effect seen in pre-menopausal women. Worse effect in post-menopausal women.

b 12% grade 3/4 toxicities, lipids not measured.

c Possible increase in second primary malignancy.

d Increased grade 3/4 anemia, hypertrigyceridemia, and febrile neutropenia.

e Significantly increased lung cancer and pulmonary disease in treated patients.

Rather, these data have provided considerable evidence about the harm of ectopic retinoid activation in humans and provided new insights into the therapeutic potential of retinoid antagonists. While a complete discussion of the results from retinoid agonist testing in humans is beyond the scope of this review, key adverse findings in oncology, immunology, and cardiometabolic disease are discussed in the context of therapeutic efforts in the pathway. Also outside the scope of this discussion, the topical use of retinoids in dermatological diseases is a broadly successful intervention for skin disorders, yet topical retinol or atRA are not absorbed systemically and thus are not discussed here (Mukherjee et al., 2006).

In contrast to the ease with which synthetic retinoid agonists were discovered, no drug-like retinoid antagonists have been introduced clinically in the century following vitamin A’s discovery. Thus, while many mechanistic studies have been conducted using pharmacologic retinoid activators, a lack of effective RAR/RXR antagonists or inhibitors of retinoic acid biosynthesis has led to an imbalance in the weight of evidence. Therefore, we focus here on genetic manipulation studies and the few tool methods used to block retinoids to examine the physiology of endogenous retinoid signaling. Illustrating the importance of genetic studies to understanding retinoid function, a seminal finding from genetic studies is that the balance between ALDH and Cyp26 enzyme expression controls retinoid activation, whereas it was once broadly presumed to be regulated by RAR isoform expression. These necessity studies have established that retinoid signaling depends on hyperlocalized retinoic acid concentrations controlled by the competing synthetic and degradation pathways (Feng et al., 2015; Heyman et al., 1992; Kane et al., 2005; Pequerul et al., 2020; Yao et al., 2018). Accordingly, this new understanding has yielded actionable insights into new approaches toward inhibiting the retinoid pathway in key diseases.

Here, we review the metabolism that controls retinoid/rexinoid signaling and the transcriptional programs mediated by retinoid signaling in normal physiology or disease, and discuss the data from clinical studies that report on retinoid pathway functions. The collective understanding of the retinoid pathway is at a key inflection point, and the goal of this review is to both caution against continued clinical testing of retinoid agonists in patient settings that may cause further harm as well as to highlight the potential for inhibitors of retinoic acid biosynthesis in well-validated human disease settings.

## Clinical and epidemiologic studies of retinoids

Retinoid therapies, including vitamin A supplementation (β-carotene, retinyl esters, and retinol), the natural pathway ligands (e.g., all-trans or 9-cis retinoic acid), and synthetic pathway ligands (e.g., tazarotene or tamibarotene), are among the most studied of all interventions in human health, yet the preponderance of evidence consistently shows these therapies to be a double-edged sword (Table 2). On the beneficial side, vitamin A supplementation in the developing world where deficiency is prevalent leads to profound improvements in early pediatric health (<5 years of age). Vitamin A supplementation in this context prevents blindness (Bowles et al., 2009) and improves gut barrier integrity through tolerance-inducing effects on the immune system (de Medeiros et al., 2018).

**Table 2.** List of FDA-approved or late-development retinoids with known common side effect profiles at pharmacologic doses

Category	Compound	Type	Receptor specificity	FDA approval	Route	Highest stage	Known adverse events
RAR agonist	Tretinoin	Natural	RARα/β/γ	APL	Oral	FDA approved	L T R B D
RAR agonist	Palovarotene	Synthetic	RARγ	Fibrodysplasia ossificans progressiva	Oral	FDA approved	D B
RAR agonist	Tamibarotene (Am80)	Synthetic	RARα	None	Oral	Phase III	L T R B D
RAR agonist	Fenretinide	Synthetic	RARα/β/γ	None	Oral	Phase III	L D V I
RAR agonist	IRX5183	Synthetic	RARα	None	Oral	Phase II	L I
RAR agonist	Amsilarotene (TAC-101)	Synthetic	RARα	None	Oral	Phase II	L
RAR agonist	Trifarotene	Synthetic	RARγ	Acne vulgaris	Topical	FDA approved	Mild D
RAR agonist	Adapalene	Synthetic	RARβ/γ	Acne vulgaris	Topical	FDA approved	Mild D
RAR agonist	Tazarotene	Synthetic	RARβ/γ	Acne vulgaris/psoriasis	Topical	FDA approved	D T
RAR agonist	Alitretinoin (9-cis RA)	Natural	RXRα/β/γ	Cutaneous Kaposi sarcoma	Topical	FDA approved	D
RXR agonist	Bexarotene	Synthetic	RXRα/β/γ	CTCL	Oral	FDA approved	L T D V H
Non-nuclear pathway	Isotretinoin (Accutane, 13-cis RA)	Natural	None	Acne vulgaris	Oral	FDA approved	D V N B
RA biosynthesis	Disulfiram	Synthetic	ALDH1a1/2 inhibitor	Alcohol aversion	Oral	FDA approved	I
RA biosynthesis	WIN18446	Synthetic	ALDH1a1,2,3	None	Oral	Phase I	Male infertility
RA biosynthesis	Talarazole	Synthetic	Cyp26 inhibitor	None	Oral	Phase II	Acutely toxic
RAR antagonist	AGN 194310	Synthetic	pan-RAR	None	Oral	Preclinical	Acutely toxic
RAR antagonist	BMS-189453	Synthetic	pan-RAR	None	Oral	Preclinical	Acutely toxic
RAR antagonist	LY2955303	Synthetic	RARγ	None	Oral	Preclinical	Acutely toxic
RAR antagonist	YCT529	Synthetic	RARα	None	Oral	Phase I	Unknown

Acitretin is not included due to unknown mechanism of action. Key for known adverse events: L: hyperlipidemia; T: teratogenic (where known); R: retinoic acid syndrome; B: bone or muscle pain; D: dermatologic symptoms such as rash; V: vision effects; I: liver damage; N: neurological symptoms; H: hypothyroidism.

Vitamin A deficiency (VAD) in adults is rare. As early as 1937, the study of natural or induced deficiency in otherwise healthy adults reported effects limited to vision loss and anecdotal evidence of decreased immunity (Jeghers, 1937). On the other hand, excess vitamin A can cause acute hypervitaminosis A or retinoic acid syndrome if too much is consumed. A classic example of retinol intoxication was observed when arctic explorers consumed polar bear liver (Leo and Lieber, 1988). This potentially lethal intoxication causes neurological (lethargy), skin (desquamation), bone (destructive osteoarthritis and fracture), and liver symptoms following acute retinol overdose while chronic but subclinical retinol overnutrition is associated with osteoporosis, lipid elevations, and insulin resistance (Corbetta et al., 2006; Penniston and Tanumihardjo, 2006; Romero et al., 1996). In animals and humans, supplementation with vitamin A at pharmacologic doses directly leads to several-fold increases in plasma atRA, 13-cis retinoic acid, and 4-oxo,13-cis retinoic acid concentrations, thus explaining how ectopic retinoid and rexinoid activation can occur following the retinol doses used in trials (Biesalski et al., 1996; Eckhoff and Nau, 1990).

Myriad studies have been conducted with synthetic atRA; the first significant finding was that ectopic atRA caused morphological changes in HL-60 leukemia cells. Subsequent clinical testing of atRA induced exceptionally potent responses in acute promyelocytic leukemia (APL) patients (Huang et al., 1988), and this treatment became the standard of care for this acute myeloid leukemia subtype. In the decade after these clinical effects were observed, it was found that the potent effects of atRA in APL were uniquely due to the t(15;17) translocation, which forms a promyelocytic-RAR α (PML-RARA) oncogenic fusion gene that is observed in 95% of APL cases and not in other cancers. Treatment with atRA or other RARα agonists causes degradation of the fusion protein, resulting in loss of the oncogenic driver and clinical remission (Yoshida et al., 1996).

Yet, before the unique etiology of APL was discovered, massive randomized clinical efforts were organized to test if retinoid supplementation could prevent other types of cancer (Table 1, upper section). This hypothesis was supported by laboratory studies that showed superphysiologic atRA concentrations (>100 nM) in vitro could cause cancer cell death or differentiation (Kusmartsev et al., 2003; Mira-Y-Lopez et al., 2000; Nefedova et al., 2007). The Carotene and Retinol Efficacy Trial (CARET), Alpha-Tocopherol, Beta-Carotene Cancer Prevention Study (ATBC), and Veterans Affairs Topical Tretinoin Chemoprevention Trial (VATTC) trials subsequently randomized 48,578 older patients, including high-risk patients such as former smokers, into retinoid or placebo arms. In a devastating result to all parties involved, all three trials unexpectedly demonstrated overwhelming evidence of harm due to retinoid treatment/supplementation. Across the CARET, ATBC, and VATTC trials, multiyear treatment caused 10–28% increases in lung cancer rates and 5–17% increases in all-cause mortality, leading to early termination of these studies (Table 1). Post-hoc analysis of the CARET trial demonstrated 52% increases in aggressive prostate cancer in the treatment arm in men who had also been using dietary supplements (Neuhouser et al., 2009). Meta-analysis of randomized controlled trials in gastrointestinal cancers further confirmed the oncogenic and increased mortality effects of vitamin A, C, and E when administered at pharmacologic doses (Bjelakovic et al., 2004).

These data should have been more than sufficient to disabuse both scientists and the general public of the notion that retinoids are beneficial in preventing cancer; however, epidemiological studies continue to report on the reduced cancer rates in individuals who consume high levels of vitamin A from sources such as fruits and vegetables (Larsson et al., 2007) and thus erroneously suggest that vitamin A consumption reduces cancer rates. It is much more likely the opposite causality exists; retrospective studies of retinoid supplementation show that long-duration supplementation with β-carotene is associated with a 3.2-fold increased risk of developing lung cancer, irrespective of gender or smoking status (Satia et al., 2009). Rather, the consumption of high vitamin A in diet may be correlated to diets rich in unprocessed foods and vegetables, which leads to a lower risk of environmentally induced lung or gastric cancers. This paradox in empirical evidence compared with the prevailing retinoid hypothesis continues at present: in vitro studies continue to be conducted that support the broadly held but incorrect hypothesis that retinoids are important cancer preventatives or anticancer therapies in solid cancers (Altucci et al., 2007; Yim et al., 2014).

In addition to the large chemoprevention trials using vitamin A, hundreds of interventional non-APL oncology trials were conducted with either native retinol, atRA, or synthetic agonists such as fenretinide and tamibarotene (Table 1). The results of these trials are stark; meta-analysis of 107 studies conducted on lung cancer alone shows no clear beneficial effect of retinol or atRA (Fritz et al., 2011). Despite this, only a few authors note the paradox between the prevailing retinoid hypothesis and the negative results from rigorously controlled trials, such as those conducted in lung cancer or head and neck cancer (Freemantle et al., 2006). Rather, the negative results are typically attributed to diverse factors such as insufficient pharmacokinetic exposure through CYP26 metabolism of atRA, unknown toxic retinol metabolites, incorrect trial populations, or epigenetic silencing of retinoid signaling (Goodman et al., 2004; Nagai and Ambinder, 2023). Among the thousands of patients enrolled in interventional non-APL cancer trials using retinoids, the only significant outcomes were the approval of the rexinoid agonist bexarotene in CTCL and the off-label use of the non-nuclear retinoid isotretinoin for neuroblastoma. Notably, randomized trials of isotretinoin could not substantiate a Food and Drug Administration (FDA) or European Medicines Agency approval in neuroblastoma due to a lack of consistent benefit (Makimoto et al., 2024), and fenretinide showed no benefit either (Children’s Oncology Group et al., 2006). This has not prevented off-label use of retinoids in multiple chemoprevention settings, such as off-label treatment with isotretinoin in non-melanoma skin cancers (Ramchatesingh et al., 2022). Notably, APL still remains the only cancer indication with an FDA-approved RAR agonist despite the hundreds to thousands of trials testing retinoid agonists in other cancer types.

It is further notable that there have not been any positive developments for synthetic RAR agonists, even though these medicinally optimized compounds have substantially improved pharmacokinetics compared with atRA by avoiding CYP26 activity. Rather, randomized trials with synthetic RAR agonists including tamibarotene and fenretinide instead showed equivocal or worse outcomes, with a notable trial of tamibarotene leading to nearly a 50% reduction in progression-free survival compared with placebo in stage 3/4 lung cancer (Table 1). Despite this, the hope that RAR agonists are effective in non-APL cancers persists (Nagai and Ambinder, 2023). Conversely, clinical data with the ALDH1a1/ALDH2 inhibitor disulfiram have accrued positive benefits, with epidemiology data demonstrating that continued use of disulfiram following a cancer diagnosis decreases mortality by 34% as compared with those who stopped use (Skrott et al., 2017), while a randomized phase 2b trial in stage 4 lung cancer showed that disulfiram exerts beneficial immunotherapeutic-like survival (Table 1), suggesting retinoid synthesis inhibitors may show beneficial effects in the same cancers that retinoid agonists exacerbate.

While the majority of retinoid interventions have been tested in dermatology and oncology, adverse event profiles from these trials provide ample evidence to support the detrimental impact of retinoids on cardiovascular health. Most significant were the findings of increased cardiovascular mortality in the CARET and ATBC trials, which is further supported by the occurrence of dose-limiting hyperlipidemias in patients on synthetic retinoids (Table 2). This latter phenotype can be so severe that statins are required to prevent acute pancreatitis in patients undergoing retinoid treatment for acne or other skin diseases (Lilley et al., 2013). There are few trials that tested cardiovascular outcomes as a primary endpoint in patients treated with synthetic retinoids (Olsen and Blomhoff, 2020); one key trial evaluated cardiometabolic parameters in patients undergoing acitretin treatment for psoriasis. During the 3 month treatment, psoriasis symptoms subsided, but a significant proportion of patients developed insulin resistance and suppression of serum high-density lipoprotein (HDL) (Corbetta et al., 2006).

Interestingly, these adverse lipid effects are observed across all classes of natural and synthetic retinoid agonists as well as specific rexinoid agonists (Table 2). Class-specific synthetic RXR agonists, such as bexarotene, demonstrate additional unique cardiometabolic outcomes, including weight gain and severe hypothyroidism (Sherman, 2003). Meanwhile, abnormalities in bone growth and resorption have long been associated with both 13-cis retinoids as well as RAR agonists, yet the insidious onset of these disorders combined with the need for microCT to quantify the changes has resulted in a limited understanding of retinoid biology in bone (McGuire and Lawson, 1987).

## The retinoid transport, stereoisomerization, and oxidation cascade

The vitamin A pathway is a multistep enzymatic and transport cascade that diverges into two non-nuclear receptor branches (the visual cycle via 11-cis retinal and the poorly understood RAR/RXR-independent 13-cis retinoic acid pathway) and two nuclear signaling pathways: (i) the canonical nuclear retinoid pathway (retinoid signaling) driven by binding of atRA to RARα/β/γ and (ii) the non-canonical rexinoid pathway driven by 9-cis retinoic acid binding to RXRα/β/γ (Fig. 1). All four branches begin with vitamin A that is transported to or stored in the liver after being processed to retinyl esters by lecithin:retinol acyltransferase (LRAT) (Hall et al., 2011b). Retinyl esters are reversibly processed to retinol, likely by multiple enzymes such as retinyl ester hydrolase or lipoprotein lipase (LPL), and retinol is loaded on apo-RBP4 in the liver (Hall et al., 2011b).

RBP4 is the main circulatory vehicle for retinol, and binding of RBP4 to stimulated by retinoic acid-6 (STRA6) expressed on specific cell types leads to diffusion of retinol through STRA6 intramembrane regions to cross the outer membrane (Chen et al., 2016). The hepatic RBP4-STRA6 transport cascade is homeostatically regulated between liver, blood, and secondary pools in adipose (Steinhoff et al., 2024); alternately, there is a non-homeostatic retinyl ester transport pathway (Fig. 1) mediated by the lipid carrier proteins (chylomicrons, very low density lipoprotein [VLDL], and albumin) that has emerged as a subsidiary source of retinoid transport in humans (Burri and Clifford, 2004; Li et al., 2014). These lipid-binding proteins bind dietary retinyl esters to transport them to the liver and other tissues. Retinoid transport via lipoproteins may be a compensatory source of vitamin A transport in carnivores such as dogs, which show much lower plasma concentrations of RBP4 (Racz et al., 2018, 2020). However, an important exception is ocular tissues, where RBP4-STRA6 mediated transport appears to be necessary (Amengual et al., 2014), thus suggesting RBP4-targeting therapies may offer unique benefits in ocular disease without causing retinol deficiency in other tissues.

The all-trans configuration for the retinoids is the default stereoisomer during dietary intake, metabolism and transport owing to the isomeric stability in the all-trans configuration (McBee et al., 2000). The location, tissue dependency, and enzymes performing the 9-cis and 13-cis stereoisomerizations are poorly understood and are inconsistently described across studies (Cheng et al., 2008; Meyskens et al., 1985). The only well-annotated stereoisomerization pathway is the visual cycle via 11-cis retinal (Farjo et al., 2009; Sahu and Maeda, 2016).

### Non-nuclear signaling: The visual cycle through 11-cis retinal

In the visual cycle that occurs only in the eye (Fig. 1), intracellular all-trans retinol is bound and modified by LRAT to form retinyl esters in retinal pigment epithelium cells. Retinyl esters are then isomerized by RPE65 to 11-cis retinyl esters that are then converted to 11-cis retinol and 11-cis retinal through the 11-cis retinol dehydrogenase pathway (e.g., CRALBP and RDH5/8/10) (Farjo et al., 2009; Sears and Palczewski, 2016). 11-cis retinal covalently binds to opsin, and photons cause photoisomerization to all-trans retinal and resultant synaptic transmission. Genetic deletion of LRAT or RPE65 predictably results in irreversible blindness (Sears and Palczewski, 2016). Notably, vitamin A deficient diets were originally used to study retinoid signaling, and since 11-cis retinal is stoichiometric to opsin protein (as opposed to retinoic acid as an amplifying nuclear ligand), the first symptom of VAD is progressive vision loss, which leads to irreversible blindness in severe cases (Li et al., 2014). Interestingly, Stargardt disease is marked by a progressive accumulation of oxidized retinal species due to a recessive loss of the ABC efflux transporter A4 (ABCA4) transporter. Tinlarebant, a small molecule inhibitor of the RBP4-retinol interaction, blocks ∼80% of retinol delivery to the eye, thereby slowing Stargardt progression while avoiding adverse effects on any other tissues (Kim and Priefer, 2021). Readers are directed to specific reviews (Sears and Palczewski, 2016) on the visual cycle for a deeper discussion.

### Non-nuclear signaling: The non-canonical 13-cis retinoic acid pathway

The second non-nuclear receptor branch of the retinoid pathway is controlled by 13-cis retinoids (isotretinoin, Accutane), the 9,13-di-cis retinoids, or other 13-cis retinoid species. The function of this pathway remains cryptic and evidence for the natural synthesis of these isomers remains scant, although it is detected at low concentrations in adult tissues (Kane et al., 2005). Neither the 9,13-cis or 13-cis isomers bind to RAR or RXR nuclear receptors at physiologically relevant concentrations (Heyman et al., 1992), and systemic administration does not result in the same clinical effects as RAR or RXR agonists (Meyskens et al., 1985). Meta-analysis showed that 13-cis retinoic acid causes dry lips and dry mouth in 100% of patients, neurologic symptoms such as headaches and fatigue in 25% of patients, and adverse mood changes in 6% of patients (Brzezinski et al., 2017). Photosensitivity, nose bleeds, and arthralgias are other common side effects of 13-cis retinoic acid (Caffery and Josephson, 1988) shared with fenretinide (Modiano et al., 1990). Importantly, 13-cis retinoic acid overdoses do not result in retinoid intoxication syndrome (Aubin et al., 1995), demonstrating that neither 13-cis retinoic acid nor its metabolites stimulate RAR signaling in humans. 13-cis retinoic acid supplementation has been extensively tested in various cancers, with possible treatment effects reported through RAR-independent effects (Meyskens et al., 1985). Isotretinoin is broadly used in dermatologic practice for cosmetic reasons as well as to prevent basal cell cancers (Peck et al., 1988). However, retinol and potentially isotretinoin have shown oncogenic effects in non-melanoma skin cancers in randomized controlled trials, although there was a lack of dose-responsiveness in these trials (Clouser et al., 2010).

### Nuclear retinoid (RAR) and rexinoid (RXR) ligand metabolism

Both the retinoid (RAR) and rexinoid (RXR) nuclear receptor pathways differentiate from the visual cycle and 13-cis pathway once intracellular retinol is bound by the CRBP1/2 proteins (Fig. 1); the CRBP proteins bind to both all-trans and 9-cis retinol/retinal but not retinoic acid stereoisomers (Kane et al., 2011; Kawaguchi et al., 2015). The binding of retinol to CRBP stabilizes, solubilizes, and lowers the apparent K_m_ for retinol or retinal oxidation by ∼3.5 fold (Wang et al., 1996). The next step of retinoid metabolism involves the oxidation of retinol to retinal performed by the retinol/alcohol dehydrogenases (RDH/ADH) or the reverse reaction performed by short-chain reductase/dehydrogenases (DHRS); both reactions utilize NAD^+^/H or NADP^+^/H cofactors. The RDH enzymes are functionally redundant and only RDH10 knockout is lethal (Duester, 2008). Some RDHs are ascribed with stereospecificity, such as RDH5, while others are promiscuous (Lidén and Eriksson, 2006). Analysis of various adult tissues demonstrates retinol concentrations of 2–20 µM (Ziouzenkova et al., 2007), which is roughly equal to the K_m_ value for the RDH enzymes (Belyaeva et al., 2008b). Upon oxidation, retinaldehyde is unstable in free form, thus the CRBP proteins further act as chaperones to prevent spontaneous reaction of the labile aldehyde (Napoli, 2000). RDH10 shows reductase as well as dehydrogenase activity and thus maintains a constant ratio of retinol to retinal without DHRS expression (Belyaeva et al., 2008a).

The final step of retinoid activation, the irreversible oxidation of retinaldehyde into retinoic acid, is performed by the aldehyde dehydrogenase 1a family (ALDH1a), including ALDH1a1, ALDH1a2, and ALDH1a3. The broader ALDH1 family, defined by >40% homology, includes ALDH2 and ALDH1b1, which are involved in ethanol metabolism, while the remaining 14 ALDH enzymes are structurally distinct enzymes that act on diverse substrates such as long-chain fatty aldehydes (ALDH3 family) or discrete metabolites such as 10-formyl THF (ALDH1L family) (Ducker et al., 2016) (Table 3). Formerly known as RALDH1, RALDH2, RALDH3, the three retinaldehyde dehydrogenases, ALDH1a1, ALDH1a2, ALDH1a3, have also been ascribed with dehydrogenase activity for short/medium-chain aliphatic aldehydes, acetaldehyde, medium chain lipid aldehydes, as well as some xenobiotics like cyclophosphamide metabolites (Koppaka et al., 2012). While these findings are often reported using recombinant protein, genetic knockout data of ALDH1a1, ALDH1a2, and ALDH1a3 suggest that these enzymes exclusively oxidize retinal in vivo (reviewed below).

**Table 3.** List of known ALDH enzymes annotated with substrate preference, genetics, and cellular localization, where known

Enzyme	Likely substrate	Tissue expression	Adult deficiency	Localization
ALDH1a1	9-Cis retinaldehyde, maybe all-trans. Cyclophosphamide metabolite	All tissues: adipose, thyroid, liver, astrocyte enriched	Healthy and resistant to obesity	Cytosol
ALDH1a2	All-trans retinaldehyde only	Reproductive tissues and immune tissues	Healthy	Cytosol
ALDH1a3	All-trans retinaldehyde	Prostate/salivary glands	Healthy	Cytosol
ALDH2	Acetaldehyde	Liver	Alcohol aversion, neurological and motor function	Mitochondria
ALDH1L1	10-Formyl-THF	Liver	Unknown, likely lethal	Cytosol
ALDH1L2	10-Formyl-THF	Pancreas	Unknown, likely lethal	Mitochondria
ALDH1b1	Acetaldehyde	All tissues	Increased steatosis	Mitochondria
ALDH3a1	Long chain lipid aldehydes, UV-based decay products, oxazaphosphorines, smoking damage	Cornea, esophagus, stomach	Viable; predisposed to cataracts	Cytosol
ALDH3a2	Long chain lipid aldehydes	All tissues	Sjogren-Larsson syndrome	Cytosol
ALDH3b1	Long chain lipid aldehydes	All tissues	Unknown	Cytosol
Aldh3b2	Long chain lipid aldehydes	Breast, esophagus, skin	Unknown	Cytosol
ALDH4a1	Glutamate semialdehyde	Kidney, liver	4-hydroxybutyric aciduria	Mitochondria
ALDH5a1	Succinate semialdehyde	All tissues	4-hydroxybutyric aciduria	Mitochondria
ALDH6a1	Methylmalonate semialdehyde	All tissues	Demyelination and methylmalonic aciduria	Mitochondria
ALDH7a1	Aminoadipic semialdehyde	All tissues	Pyridoxine-dependent epilepsy	Mixed
ALDH8a1	2-Aminomuconate semialdehyde, potential 9-cis retinal	Kidney, liver	Unknown	Cytosol
ALDH9a1	GABA	All tissues	Unknown	Cytosol
ALDH16a1	Non-catalytic	Low basal expression	Unknown	Cytosol
ALDH18a1	Pyrroline-5-carboxylate	All tissues	Cutis laxia	Mitochondria

Although the retinol–retinal conversion is reversible, retinaldehyde concentrations in tissues are 10-fold lower than retinol at 0.2–0.8 µM (Ziouzenkova et al., 2007), which is well below the K_m_ for each ALDH1a enzyme (Pequerul et al., 2020). Meanwhile, retinoic acid concentrations in ALDH1a-positive tissues are 100-fold lower than retinaldehyde at ∼5 nM (Kane et al., 2005). The K_cat_ values for retinaldehyde for each ALDH1a isozyme are exceptionally low at 10−20 min^−1^ (Pequerul et al., 2020). Despite the large enthalpic change upon retinal oxidation, the slow kinetic rate for the ALDH1a enzymes and the tissue concentrations of retinal versus retinoic acid demonstrate that ALDH1a enzyme concentration functionally controls retinoid signaling.

Kinetic characterization of the three retinal dehydrogenase enzymes (ALDH1a1, 1a2, and 1a3) reveals that ALDH1a1 is the chief enzyme responsible for 9-cis retinal oxidation (Labrecque et al., 1995; Paterson et al., 2013). On the other hand, ALDH1a2 shows much lower k_cat_/K_m_ for 9-cis retinal while ALDH1a3 shows no conversion of 9-cis (Pequerul et al., 2020). Meanwhile, k_cat_/K_m_ of the all-trans retinal is three- and fivefold greater for ALDH1a2 and ALDH1a3 compared with ALDH1a1, respectively. ALDH1a1 is strongly inhibited by divalent cations (Pequerul et al., 2020) as well as SIRT2 acetylation leading to degradation (Zhao et al., 2014). Genetic knockout studies have revealed that ALDH1a1 knockout mice are viable with no changes in liver atRA while differences in retinol precursors are observed (Fan et al., 2003). ALDH1a1 knockout mice do not replicate the effects of either RARα/β/γ or ALDH1a2/ALDH1a3 knockouts (Shortall et al., 2021), supporting a predominant role for ALDH1a1 in 9-cis retinoic acid synthesis in the adipose tissue or melanocytes (Paterson et al., 2013). However, as 9-cis retinoic acid activates the RAR receptors and ALDH1a1 also oxidizes all-trans species, this does not preclude its role in all-trans retinal oxidation in tissues with high concentrations such as the testes or liver (Topping and Griswold, 2022).

Retinoic acid acts intracellularly or diffuses as a paracrine signal; in both cases, it appears to require binding to CRABP (I/II) proteins expressed by target cells. These proteins shuttle cytoplasmic retinoic acid to the nucleus to bind the nuclear receptors (Isoherranen and Zhong, 2019). Whereas the ALDH1a enzymes are inefficient, K_d_ values for retinoic acid to the RAR nuclear receptors are exceptionally avid at 4–15 nM (Heyman et al., 1992), thus RAR signaling is exquisitely sensitive to small amounts of atRA produced by ALDH1a activity. Further supporting the idea that retinoic acid biosynthesis is the key determinant of retinoid signaling, the RAR/RXR receptors are broadly expressed in both developmental and adult tissues (Arfaoui et al., 2013; Harada et al., 1995; Mollard et al., 2000), while ALDH1a2 is restricted to immune cells and reproductive tissues and ALDH1a3 protein is not expressed in healthy adult tissues (Uhlén et al., 2015). This notion that retinoid activation is controlled by ALDH1a expression is further supported by recent studies (Feng et al., 2015; Yao et al., 2018).

The retinoid and rexinoid pathways are activated depending on whether all-trans or 9-cis retinoic acid is generated. atRA only binds RAR receptors and shows no affinity for RXR receptors (Heyman et al., 1992), while 9-cis retinoic acid binds both RXR receptors as well as RAR receptors, albeit interactions with RAR receptors have 1.6- to 12-fold lower affinity (Allenby et al., 1994; Heyman et al., 1992). The natural source of 9-cis isomerization remains a contentious issue as it is not synthesized in cultured cells, while other studies identify 9-cis in developing embryos, liver, kidney, and pancreas (Cheng et al., 2008; Heyman et al., 1992; Kane, 2012). Studies administering dietary 9-cis β-carotene demonstrate that 9-cis retinoids are rapidly converted to all-trans isomers before entering the bloodstream, therefore a specific biochemical pathway to produce 9-cis isomers intracellularly must exist (You et al., 1996). Some studies suggest that 9-cis retinoic acid can be generated from the direct isomerization of atRA in the liver (Urbach and Rando, 1994). However, isomerization likely occurs on the ester or alcohol in the hepatobiliary system (Kane, 2012). Compared with the all-trans and 9-cis stereoisomers, 11-cis and 13-cis isomers show >40-fold lower affinity for RXR or RAR receptors and are not sources of nuclear receptor activity at endogenous levels (Heyman et al., 1992), yet may have mixed RAR-like effects at pharmacologic doses (Marsden et al., 1984). Supporting the hypothesis that ALDH1a1 drives rexinoid activation while ALDH1a2/1a3 drives retinoid activation, ALDH1a1 knockout mice exhibit increased brown fat synthesis and obesity resistance (Kiefer et al., 2012), which phenocopies RXRγ but not RAR knockout (Haugen et al., 2004). Alternately, either ALDH1a2 or ALDH1a3 knockout leads to perinatal death due to atRA deficiency that can be rescued through RAR activation but not RXR activation (Dupé et al., 2003; Mic et al., 2003). Upon generation of all-trans or 9-cis retinoic acid from retinal isomers, retinoic acid is chemically stable but is rapidly degraded by the CYP26 enzymes CYP26A1, CYP26B1, and CYP26C1. Chronic treatment of humans with retinoic acid causes CYP26 induction and rapid turnover of atRA (Hall et al., 2011b). The CYP26 enzymes are also instrumental in generating morphogenic gradients of atRA in the developing embryo, with Cyp26 knockout in mice causing developmental defects that can be rescued by Aldh1a2 knockout (Niederreither et al., 2002). CYP26 enzymes metabolize all-trans, 9-cis, and 13-cis stereoisomers as well as the retinoic acid metabolites 4-oxo-RA and 4-hydroxy-RA (Isoherranen and Zhong, 2019). Whereas 4-hydroxy-RA and 4-oxo-RA were once presumed to activate retinoid signaling, this has since been disproven (Mic and Duester, 2003; Niederreither et al., 2002), rather, this is the key step in inactivating retinoid signaling and initiating metabolic elimination.

## Functional importance of retinoic acid signaling

The RAR and RXR nuclear receptors show remarkable complexity with three genes for each and sub-isoforms defined by differing N-terminal A/B domains driven by alternative splicing (Cheng et al., 2008). RAR receptors form obligate heterodimers with RXR, and these pairs present diversely across tissues, with RAR activity dominant over RXR activity (Kane, 2012). This diversity in binding leads to disparate transcriptional outputs depending on the isoform and context, and these transcriptional characterizations are always evolving (Duester, 2008). Importantly, while many studies of RAR transcriptional networks have been performed, these often employ synthetic retinoid agonism in cells that are not natively exposed to atRA and thus are not discussed here.

### RAR transcriptional networks in normal physiology

Both retinoid and rexinoid nuclear receptor activation via retinoids are dispensable from postnatal development to healthy adult physiology. These critical data derive from whole-body genetic knockouts of ALDH1a1, ALDH1a2, or ALDH1a3. Germline knockout of ALDH1a2 or ALDH1a3 causes embryonic lethality, but ALDH1a3 knockout could be rescued by maternal injections of atRA between embryonic day 9 and day 11, resulting in live births and healthy, fertile ALDH1a3^−/−^ adult mice (Dupé et al., 2003). In the case of ALDH1a2 knockout, injections during the same time rescued some features of development but did not result in live births (Mic and Duester, 2003). Meanwhile, inducible knockout of ALDH1a2 or compound ALDH1a1/1a2/1a3 knockout is well-tolerated in adult mice, with multiple independent labs reporting the generation and viability of these mice (Kumar et al., 2017; Topping and Griswold, 2022). ALDH1a1 knockouts are not embryonically lethal, but are fertile and are furthermore resistant to high-fat diet–induced obesity (Kiefer et al., 2012). Compound whole-body ALDH1a1 and Sertoli cell–specific ALDH1a2 knockout in mice blocks spermatogenesis, which can be rescued by ectopic atRA (Topping and Griswold, 2022). Importantly, these genetic studies have been performed in sterile breeding facilities with few immune challenges and in younger mice, thus the susceptibility of ALDH1a knockout mice to immune challenges or aging-related diseases has been overlooked. Single and compound ALDH knockouts still need to be studied in many disease contexts where clinical data suggests a role.

Human trials with WIN18446, a pan-ALDH1 family covalent inhibitor, reported that only spermatogenesis is affected in healthy volunteers (Heller et al., 1961). This is supported by human and animal data showing that vitamin A–deficient diets and pan-ALDH1 inhibitors result in mild phenotypes in adults such as mild leukocytosis and enhanced T cell proliferation (Wiedermann et al., 1996), reversible arrest of spermatogenesis (Amory, 2016), gastrointestinal inflammation (Heller et al., 1961), and potentially improved lipid (Munson et al., 2004) or glucose (Nagai et al., 2009) control. Disulfiram (Antabuse), a potent ALDH1a1/ALDH2 covalent inhibitor, has been used extensively in humans for >50 years as an alcohol aversion agent with broad evidence showing that inhibition of either enzyme has little impact on health (Johansson, 1992).

In parallel to ALDH1a genetic studies, RAR/RXR receptor knockout demonstrates severe developmental defects (Berenguer et al., 2018; Dupé et al., 2003; Ghyselinck et al., 1997), while in healthy adults, the impact of RAR signaling appears to be restricted to controlling immune populations and initiating spermatogenesis (Hall et al., 2011b; Hogarth et al., 2011). RAR transcriptional activity is well characterized, often through the use of ectopic retinoid agonists, while RXR receptors remain poorly characterized even though several datasets are available with bexarotene treatment. Beyond serving as obligate heterodimers for nuclear RAR receptors, RXR receptors are permissive partners in heterodimer complexes with the liver X receptor, farnesoid X receptor, and peroxisome proliferator–activated receptor (PPAR) (Desvergne, 2007). Interestingly, compound knockouts of RXR (α −/+, β −/−, γ −/−) are healthy with no overt phenotypes (Krezel et al., 1996). Meanwhile, triple knockouts of RAR α/β/γ eliminate retinoid signaling and cause early lethality (Laursen and Gudas, 2018). Single RARα or RARγ knockout is perinatal lethal (Labrecque et al., 1998) and RARβ knockouts show growth retardation (Ghyselinck et al., 1997). Thus, each RAR receptor has unique functions, at least in development, while only a single RXR allele is needed across nuclear receptor signaling pathways. For further discussion of RAR/RXR transcription, readers are directed to in-depth reviews on the mechanistic impact of RAR signaling in spermatogenesis (Hogarth et al., 2011), development (Duester, 2008), and dermatological applications using topical retinoids (Duvic et al., 2000; Kane, 2012).

### Retinoid signaling is a master immune regulator

**Figure 2.**
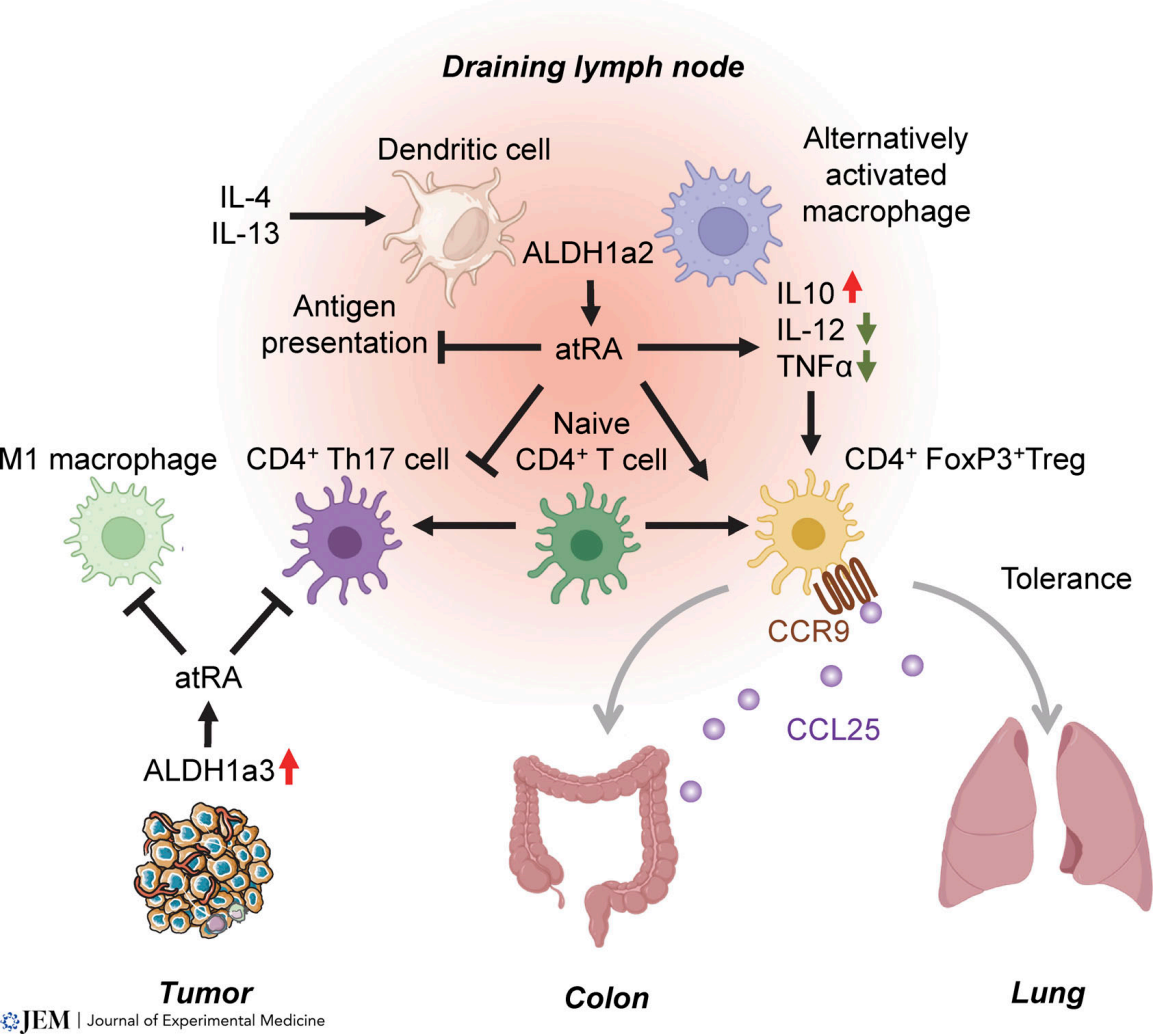
**Retinoid signaling is a master regulator of immune tolerance in mucosal tissues and tumors.** IL-4 and IL-13 induce ALDH1a2 expression in tolerogenic DCs or alternatively activated macrophages of either the lymph nodes or tertiary lymphatic structures, which regulates both myeloid and lymphoid immunity through generation of atRA. ALDH1a2-produced atRA is an autocrine regulator of many myeloid cell functions through suppression of inflammatory cytokines, including IL-12 and TNFα, and stimulation of IL-10 production. The paracrine effect of atRA suppresses CD4^+^ T_h_17 cell differentiation and instead biases differentiation toward CD4^+^ FoxP3^+^ T_reg_ cells. Imprinting of CCR9 onto the surface of trafficking lymphocytes provides tissue specificity for tolerance-inducing Treg migration toward organs with high expression of the CCR9 ligand CCL25, such as the colon. Tumors opportunistically express ALDH1a2 or ALDH1a3 to hijack the normal tolerizing effects of atRA and directly suppress effector T cells through paracrine activity of atRA. Paracrine atRA also suppresses antigen presentation and phagocytosis of M1 macrophages and instead pushes monocyte differentiation toward immature, M2-like macrophages. Illustration created in part with Biorender.com.

Epidemiologic data from natural deprivation settings have established that VAD is marked by a loss of T_h_2 signaling and T cell homing to the gut, while vitamin A supplementation alleviates gastrointestinal and airway inflammation (Iwata, 2009). Retinoid nuclear signaling influences immune populations from early hematopoiesis (Evans, 2005) through the differentiation and activity of dendritic cells (DCs), macrophages, and lymphocytes. Direct clinical evidence for the role of retinoids in immune tolerance derives from the potency of atRA in causing remission of immune thrombocytopenia and psoriasis (Table 1). VAD in rodents leads to systemic inflammation and increased reactivity of both T cells and mast cells (Wiedermann et al., 1996). The summation of evidence across studies suggests that retinoid signaling induces tolerance in type 1 inflammation while it is proinflammatory in type 2 inflammation (Fig. 2); however, there are several caveats to this paradigm.

The mechanistic basis for retinoid-controlled immune tolerance was identified when TGF-β and atRA secreted from tolerogenic DCs were found to differentiate regulatory T cells (T_reg_) from naïve CD4^+^ T cells (Galvin et al., 2013; Maldonado and von Andrian, 2010; Mucida et al., 2007). Later studies suggested that atRA indirectly promoted T_regs_ by suppressing CD4 T_h_1 cell proliferation and survival (Hill et al., 2008). Other studies showed that atRA induced Arg-1 and iNOS in DCs to suppress T cell proliferation (Bhatt et al., 2014) or that atRA downregulated IL-12 while increasing IL-10 in human monocytes to generate T_h_2 T cells or T_regs_ (Jin et al., 2010). Monocyte-to-macrophage differentiation is stimulated by atRA as exogenous atRA supplementation shifts cells from an M1 to an M2 lineage and inhibits macrophage-mediated immunity (Vellozo et al., 2017). Treatment with AGN194310, a pan RAR antagonist, expands granulocytes in vivo without affecting terminal T cell maturation (Walkley et al., 2002).

Studies have consistently demonstrated that ALDH1a2 generates atRA in the immune system to inhibit inflammatory T_h_17 maturation from naïve T cells (T_n_) while it instructs T_n_ maturation toward FoxP3^+^, CTLA-expressing T_reg_ cells (Mucida et al., 2007). ALDH1a2 in mucosal tissues is either expressed by regulatory CD103^+^ or plasmacytoid DCs (Lee et al., 2012; Zhu et al., 2013). ALDH1a2 is also induced in bone marrow–derived DCs (BMDCs) by integrin and TLR binding to promote gut immune tolerance (Yokota-Nakatsuma et al., 2016). Loss of ALDH1a2 expression via the CD11c-specific deletion of *Rbtj* led to colitis as inducible T_reg_ differentiation was lost (Zaman et al., 2017). Loss of CD55 (decay accelerating factor) has also been associated with irritable bowel syndrome, and this was shown to occur via the loss of ALDH1a2 expression in DCs (Strainic et al., 2019). Outside of the gut-associated lymphoid tissue, skin draining CD103^−^ regulatory, plasmacytoid CD8α^+^, or splenic DCs express ALDH1a2 to enforce T_reg_ induction (Guilliams et al., 2010; Manicassamy et al., 2009; Lombardi et al., 2012). Engineering of DCs to overexpress ALDH1a2 suppresses experimentally induced colitis (Xu et al., 2019).

Recent studies have implicated ALDH1a2 in the immunoregulatory properties of monocyte-derived macrophages, with tolerogenic macrophage-like CD206^+^ cells expressing ALDH1a2 (Yang et al., 2020), lung resident macrophages inducing ALDH1a2 to promote T_reg_ maturation leading to airway tolerance (Soroosh et al., 2013), and IL-4R–stimulated monocytes differentiating into alternatively activated macrophages that induce T_reg_ formation via ALDH1a2 activity (Gundra et al., 2014). Lamia propria macrophages also secrete retinoic acid via ALDH1a2 to induce T_regs_ (Denning et al., 2007).

On the other hand, there are two settings that show a proinflammatory mechanism of retinoid signaling. In type 2 inflammation, ALDH1a2 in human basophils mediates an allergic response (Spiegl et al., 2008), or ALDH1a2 induction in alternatively activated macrophages helps mount a T_h_2 response during helminth infection (Broadhurst et al., 2012; Kannan et al., 2016). Stimulation of DCs with GM-CSF induces ALDH1a2 to produce atRA, which may promote antibacterial activity (Kim et al., 2019). In the second setting of alloreactivity, two studies have implicated ALDH1a2 activity in promoting acute graft-vs-host disease (aGVHD) with radiation-inducing ALDH1a2 to exacerbate aGVHD (Zheng et al., 2020) and ALDH1a2 deletion alleviating aGVHD (Thangavelu et al., 2019). This is an interesting scenario where the normal tolerance-inducing properties of retinoid signaling become proinflammatory: in aGVHD, ALDH1a2 expressed by host DCs imprints the mucosal homing receptors α4β7 and CCR9 on donor lymphocytes to migrate to the colon (Aoyama et al., 2013; Thangavelu et al., 2019; Zheng et al., 2020). This was also supported by another study on mucosal infection and vaccination that identified a proinflammatory mechanism for RAR signaling through imprinting of the mucosal trafficking receptors (Hall et al., 2011a). In both cases, imprinting of normally tolerogenic lymphocytes to a site with high allo- or autoreactivity may provide ample stimulus to mount an inflammatory response. A recent study showed that DCs in lymph nodes shared between the pancreas and duodenum utilize ALDH1a2 to imprint CCR9 on homing lymphocytes, thus suggesting how ALDH1a2 may be inflammatory in certain autoimmune diseases of the gut (Brown et al., 2023), potentially explaining recent positive impacts of WIN18446 in inflammatory bowel disease models (Seamons et al., 2020).

### Retinoid signaling in cancer is a paracrine, immunosuppressive pathway

The application of the Aldefluor assay was a critical step in understanding the prevalence of ALDH1a activity in cancer. Studies showed that Aldefluor-positive cancer cells were more tumorigenic, predicted worse clinical outcomes (Ginestier et al., 2007), and were detected across many cancer types (Devalaraja et al., 2020; Marcato et al., 2011). Since, ALDH1a3 has been identified as the most common ALDH1a isoform expressed across cancers and which functionally promotes cancer growth, therapy resistance or metastasis in melanoma (Luo et al., 2012; Pérez-Alea et al., 2017), glioma or glioblastoma (Cheng et al., 2016; Flahaut et al., 2016; Mao et al., 2013; Ni et al., 2019; Sullivan et al., 2017; Wu et al., 2018; Zhang et al., 2015), lung cancer (Rebollido-Rios et al., 2020; Shao et al., 2014; Yun et al., 2018), pancreatic cancer (Jia et al., 2013; Kim et al., 2014), colon cancer and thyroid cancer (Golubovskaya et al., 2015; Lee et al., 2019), mesothelioma (Canino et al., 2015; Cortes-Dericks et al., 2014; di Martino et al., 2018), prostate cancer (Casanova-Salas et al., 2015; Wang et al., 2017), breast cancer (Kashii-Magaribuchi et al., 2016; Marcato et al., 2015; Thomas et al., 2016; Yamashita et al., 2020), head and neck cancer (Gui et al., 2020), and cholangiocarcinoma (Chen et al., 2016).

Complementing tumor ALDH1a3, ALDH1a2 is found in the cancer stroma, such as the immunoregulatory mucosal myeloid regulatory DCs found in lung cancer patients (Maier et al., 2020) or the M2 macrophages in glioblastoma that promote tumor tolerance (Sanders et al., 2021). Interestingly, *ALDH1a2* RNA amplification is necessary for LMO1-fusion T cell acute lymphoblastic leukemia (T-ALL) transformation (Ono et al., 1998; Zhang et al., 2021), showing that atRA has unique autocrine cancer effects in this specific T cell leukemia. Earlier studies also showed that ALDH1a2 was amplified in nearly all T-ALL patients, including TAL1-negative patients, suggesting a critical need for ALDH1a2 activity (Palomero et al., 2006). Other studies showed that inhibition of ALDH1a2 reduces T_regs_ in lymphomas and melanomas, leading to decreased tumor size (Hong et al., 2015). ALDH1a1 has been implicated as an immunotherapy target in liver cancer through similar mechanisms as ALDH1a3 in sarcomas (Yu et al., 2024), suggesting that the immunotherapeutic-like effects of disulfiram in lung cancer may be due to ALDH1a1.

These findings directly contradict the prominent retinoid hypothesis of tumor suppression and should give pause to researchers who continue to hypothesize that retinoic acid is an important tumor suppressor. Why would cancers specifically amplify the retinoid synthesis pathway if they were sensitive to retinoic acid–induced growth suppression? This paradox has led many researchers to suggest non-retinoid mechanisms for ALDH1a enzymes, yet other studies have more recently shown that atRA accelerates tumor growth when administered to tumors that express the ALDH enzymes (Heine et al., 2017). A groundbreaking study from Devalaraja and colleagues provided a unifying theory by showing that tumor-expressed ALDH1a3 generates retinoic acid as a paracrine factor to activate tumor-associated macrophages and suppress CD4 T cell activity (Devalaraja et al., 2020). This study complements the finding that ALDH1a3 is significantly enriched in anti PD-1, anti-CTLA4 non-responders (Liu et al., 2019). The paracrine immune mechanism of atRA also explains why ectopic expression of ALDH1a2 or ALDH1a3 in ALDH-negative cancer cells is often growth suppressive (Kim et al., 2005). These cells would never have been exposed to atRA during tumorigenesis, and though the tumor cells may be sensitive to retinoid agonism in vitro, data have demonstrated that neither native nor synthetic RAR agonists are effective in treating solid cancer patients (Table 1).

There were several early studies to suggest that the retinoid hypothesis in cancer was flawed, but without a unified understanding of retinoid activity, these studies were not informative to clinical testing. For instance, epithelial ER^+^ breast cancer cell lines T47D and MCF7 express the RAR nuclear receptors but not the ALDH1a enzymes, and thus atRA but not retinol causes their growth arrest. Meanwhile, more aggressive ER-negative, ALDH1a3-positive cell lines such as MDA-MB-468 could synthesize atRA from retinol, but atRA does not affect their growth (Mira-Y-Lopez et al., 2000). Multiple studies suggested direct cytotoxic effects of high-dose atRA in xenograft tumors (Nguyen et al., 2016), yet this was not observed in clinical patients (Smith et al., 1992). When it was found that atRA treatment was not therapeutic in multiple syngeneic tumors (Kusmartsev et al., 2003), other studies attempted to rationalize the difference in atRA responsiveness through differential nuclear receptor partitioning (Schug et al., 2007) rather than looking for the simple alternative hypothesis.

## ALDH1a1 and ALDH1a3 drive several lipid-related metabolic disorders

The bulk of our knowledge concerning retinoid signaling in human cardiometabolic diseases is restricted to the adverse effects of retinoid agonists in human trials for cancer and dermatology (Table 1). Across these clinical studies, retinoid agonists are well-established to drive dyslipidemias, with 50% of patients experiencing grade 3 or grade 4 dyslipidemias in some trials (Table 1). There are comparable data with the RXR agonist bexarotene, where it is known to cause hypertriglyceridemia (79% of patients), hypercholesterolemia (48%), and hypothyroidism (40%) in CTCL patients (Assaf et al., 2006), along with severe hypothyroidism and weight gain in other studies (Makita et al., 2019).

Interestingly, most tissues are known to absorb systemically administered atRA, yet the testes and pancreas are the only two tissues known to specifically exclude absorption of systemic atRA (Kurlandsky et al., 1995). Meanwhile, retinyl esters are stored broadly throughout the body, with major reservoirs in the liver, pancreas, and adipose tissue (Brun et al., 2016). This phenomenon suggests two insights: (1) the pancreas and testes have evolved a protective mechanism to isolate retinoid signaling from other tissues; and (2) studies administering systemic atRA and potentially other retinoid agonists will not directly activate pancreatic or testicular retinoid signaling (Brun et al., 2016), indicating that data from diabetes and fertility studies may need to be reassessed.

**Figure 3.**
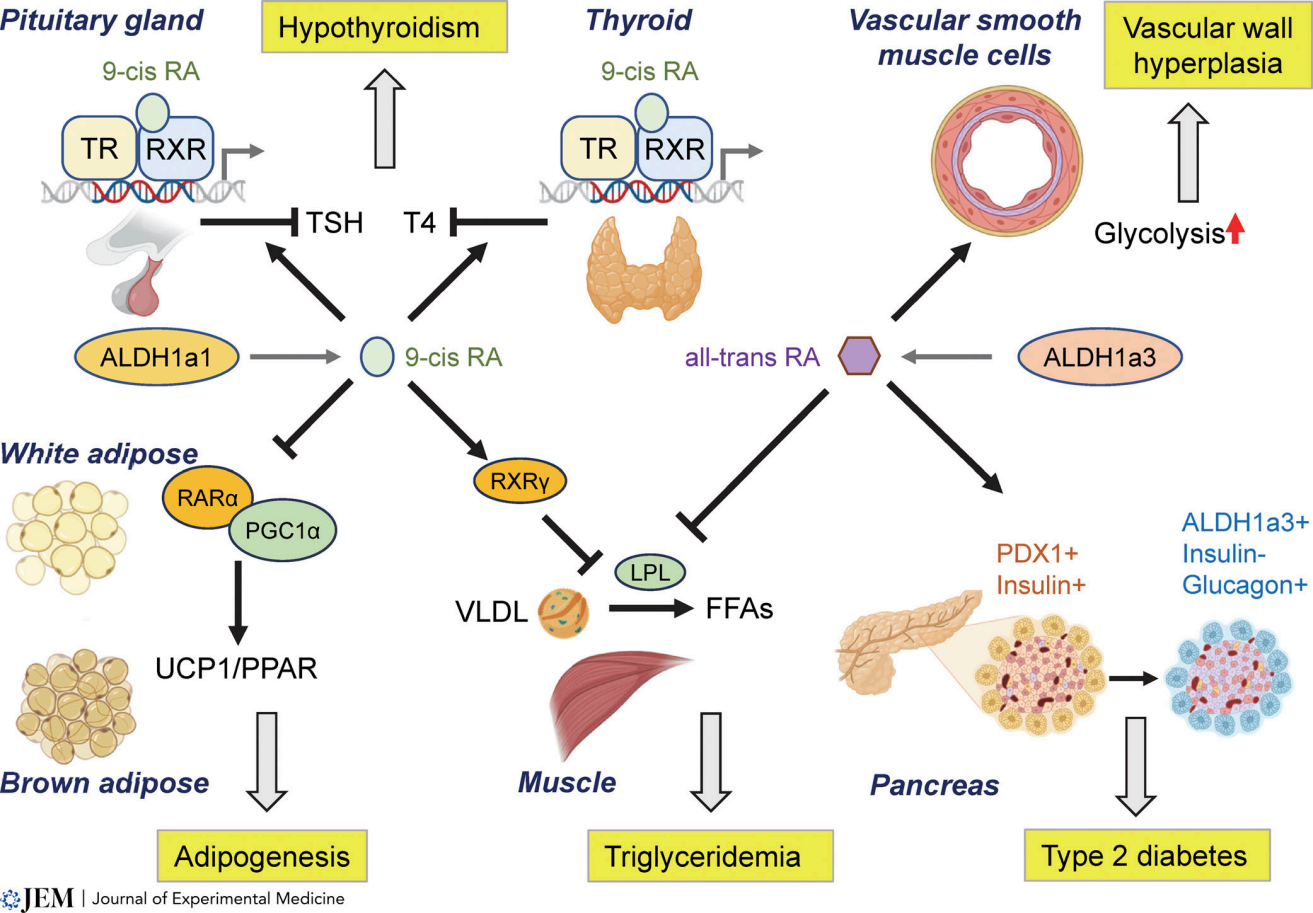
**Retinoid and rexinoid signaling regulate metabolism toward lipogenesis.** ALDH1a1 is expressed in adipose and hepatic cells to generate the rexinoid ligand, 9-cis retinoic acid. In the thyroid, 9-cis retinoic acid (RA) binding to the RXR-thyroid receptor (TR) heterodimer represses the production of free T4 while in the pituitary, 9-cis retinoic acid suppresses thyroid stimulating hormone (TSH) mRNA and protein, leading to hypothyroidism. In white adipose tissue, ALDH1a1 expression correlates with white adipocyte mass and increases white adipogenesis by blocking thermogenic adipose programs driven by PGC1α-driven transcription of PPAR and UCP1. Both ALDH1a1 and ALDH1a3 produced retinoids directly suppress LPL expression in muscle tissues, thus preventing cleavage of triglycerides in VLDLs into free-fatty acids available for use in muscle tissues. ALDH1a3 is induced in diseased or injured tissues such as exhausted pancreatic β-islets where it drives a dedifferentiation program, thus preventing glucose-responsive insulin secretion. ALDH1a3 is also expressed in proliferative smooth muscle cells, and ALDH1a3 regulation of glycolysis leads to SMC proliferation and pulmonary hypertension or neointimal hyperplasia. Illustration created in part with Biorender.com.

In type 2 diabetes and vascular proliferation diseases, ALDH1a3 has become a standard marker of cellular dedifferentiation (Fig. 3), particularly in failing pancreatic β cells of both patients and mouse models (Davey et al., 2020; Kim-Muller et al., 2016; Wang et al., 2020). Additional research indicates that ALDH1a3 expression directly reduces insulin secretion by pancreatic β cells while increasing glucagon secretion (Shimamura et al., 2010). This was paralleled by the findings that an islet β cell–specific dominant-negative RXR receptor prevented the diabetic phenotype (Miyazaki et al., 2010) while increasing 9-cis retinoic acid concentrations prevents glucose-mediated insulin secretion (Kane et al., 2010). Treatment of type 2 diabetic rats with high doses of disulfiram also reverses diabetes progression by restoring insulin homeostasis (Nagai et al., 2009). miR-483 was described as a protective factor in type 2 diabetes by repressing ALDH1a3 and knockout of miR-483 led to β cell dedifferentiation (Wang et al., 2021).

We have validated ALDH1a3 as an essential driver of pancreatic β cell loss whose inhibition restores insulin secretion in late-stage type 2 diabetes. In this study, conditional knockout of ALDH1a3 in β cells reverses dedifferentiation in models of type 2 diabetes, and treatment with a specific ALDH1a3 inhibitor causes the pancreatic islets to functionally regenerate, which is dependent on the loss of retinoid signaling. Importantly, the transcriptional networks driving this phenotype were characterized in the context of ALDH1a3 loss rather than retinoid agonist treatment (Son et al., 2023). ALDH1a3 has also been implicated in a driving role in neointimal hyperplasia and pulmonary arterial hypertension (PAH) where it is induced in both human tissues and mouse models (Xie et al., 2019). A recent study identified ALDH1a3 as the top differentially induced gene in human PAH smooth muscle cells and smooth muscle cell-specific deletion of ALDH1a3 did not affect normal mice but alleviated chronic hypoxia-induced PAH in male mice (Li et al., 2021).

The role of retinoids in obesity has rapidly become a major focus given the success of GLP-1R agonists. While there are several preclinical studies suggesting retinoid activation either promotes weight loss or causes weight gain, an FDA-listed side effect of either tretinoin or bexarotene therapy is hypothyroidism and weight gain. In healthy rats, atRA increases liver weights and VLDL levels as well as insulin resistance (Krupková et al., 2009). Well-considered genetic studies have since elucidated this biology, including the independently replicated findings that either ALDH1a1 or RXR genetic knockout leads to obesity resistance and that pharmacologic treatments with either WIN18446 or disulfiram similarly reverse obesity. In a series of high-profile studies, disulfiram was found to promote weight loss in obese animals while not reducing food intake or lean muscle mass (Bernier et al., 2020). This was ascribed to increased energy expenditure in adipocytes and the prevention of adipogenesis. WIN18446 has also been shown to be a potent antiobesity treatment due to its inhibition of ALDH1a1, although hepatic lipidosis is a side effect of the drug that is not witnessed in ALDH1a1 knockout mice (Haenisch et al., 2018) or disulfiram-treated mice (Bernier et al., 2020).

There are few studies of the cardiometabolic effects of either disulfiram or WIN18446 in non-rodent models. Treating cats with WIN18446 over 76 days resulted in effective spermatogenic arrest and also marked decreases in cholesterol, alanine aminotransferase, and aspartate aminotransferase, suggesting that WIN18446 resulted in beneficial vascular and hepatic effects (Munson et al., 2004). While the side effect profile of either disulfiram or WIN18446 prevents dosing in humans for obesity, testing retinoid inhibitors in obese, non-human primate models would provide considerable insights into the role of the pathway in cardiometabolic diseases.

Independent of ALDH1a1/1a3, RBP4 has also become a validated marker in type 2 diabetes. RBP4 is characterized as a prodiabetes cytokine whose knockout promotes diabetes resistance but also vision loss (Yang et al., 2005). RBP4 is also an adipokine marker elevated in both chronic kidney disease (Jing et al., 2016) and chronic artery disease (Lambadiari et al., 2014). This suggests that higher retinoid signaling in these tissues promoted by ALDH1a1/1a3 expression may increase RBP4-mediated retinol delivery to adipose or pancreas.

### A perspective on antagonizing the retinoid pathway

Despite the importance of clinical findings that retinoid activation drives the progression of solid cancers as well as metabolic syndromes in humans, antagonists of the retinoid pathway have not been approved. The earliest attempts to antagonize this pathway utilized VAD, pan-ALDH covalent inhibitors such as WIN18446, or non-selective cysteine covalent modifiers like disulfiram (Antabuse). Despite the effectiveness of WIN18446 across several potential indications in preclinical studies, further attempts to commercialize this were abandoned due to its lack of ALDH isoform selectivity leading to inhibition of ALDH2 (Amory et al., 2011) as well as hepatic lipidosis, which is one of the off-target concerns (Haenisch et al., 2018). Meanwhile, disulfiram exhibits many negative side effects, including ALDH2 inhibition (Table 2).

Most pharmaceutical drug development efforts have focused on the RAR receptors, potentially due to the inferred complexity of retinoic acid metabolism. Three lead compounds were reported with RAR antagonist activity (BMS189453, AGN194310, and LY2955303), yet the BMS189453 and LY2955303 compounds exhibit unacceptable hepatic and testicular toxicity (Hughes et al., 2016; Schulze et al., 2001) while toxic effects of AGN194310 (VTP194310, IRX4310) were not published. More recently, YCT529, which is a modified scaffold of BMS189453, has been developed with no reported toxicity and high RARα selectivity (Al Noman et al., 2023). Single-dose escalation in a phase 1a male contraceptive trial is completed in healthy volunteers, yet pharmacologic activity in blocking spermatogenesis or other retinoid effects has not yet been tested.

As evidenced by human trials with WIN18446 and broad usage of disulfiram before its use was discontinued, inhibition of ALDH1a enzymes in adults is well-tolerated in humans. This is further supported by epidemiologic evidence: in humans, homozygous hypomorphic mutations in ALDH1a3 are associated with small-eye disease in consanguineous families that carry multiple homozygous mutations, yet siblings bearing the same ALDH1a3 mutations showed no adverse developmental effects (Plaisancié et al., 2016). Studies in mice have shown that ALDH1a3 is specifically repressed in certain developmental tissues to prevent retinoid signaling while it is critical in others during fetal development (Yao et al., 2018). Postnatal, ALDH1a3 is dispensable to the developing ovary (Minkina et al., 2017), its mRNA is expressed only in the prostate and salivary glands of healthy human adults (Wang et al., 2017), and studies have not demonstrated a functional role for ALDH1a3 in healthy adults (Plaisancié et al., 2016). ALDH1a3 mRNA is marginally expressed in reproductive tissues, but ALDH1a3 knockout mice are fertile (Dupé et al., 2003). ALDH1a2 parallels the role of ALDH1a3 in development; early observations of knockout mice revealed that ALDH1a2 is necessary for embryonic survival, and inducible whole-body knockout is well-tolerated (Berenguer et al., 2018; Niederreither et al., 1999). In parallel, either RXR receptor or ALDH1a1 knockout appears to show no deleterious effects and rather protects against high-fat diet-induced obesity (Haugen et al., 2004; Kiefer et al., 2012). Synthesizing this data, inhibitors of ALDH1a1 or ALDH1a3 in the cardiometabolic context would likely show few side effects and thus offer a beneficial profile. Meanwhile, ALDH1a2 inhibitors may show either gut or airway inflammation; however, their testing for either cancer, autoimmune disease, or aGVHD would be accommodative of such side effects. The design of second-generation RAR inhibitors is more difficult as many of these compounds exhibit diverse agonist, antagonist, and inverse agonist properties.

Drug discovery in cancer research has thus far been applied to ALDH1a1, ALDH1a3, and ALDH3a1 while drug discovery in substance abuse has focused on ALDH2, and discovery in the male contraceptive space has focused on ALDH1a1/a2 or RARα (Koppaka et al., 2012). The majority of drug discovery work has focused on covalent inhibitors and slow turnover substrates targeting the broad ALDH family; these include Win18446 (Amory et al., 2011), disulfiram (Moreb et al., 2012), citral (Pequerul et al., 2020), DIMATE (Pérez-Alea et al., 2017), Aldi-1 (Khanna et al., 2011), and Bay-11-7085 (Yasgar et al., 2017). Work with nearly all of these covalent inhibitors show IC50 values in the 5–100 μM range (Cheng et al., 2016; Thomas et al., 2016), and independent review suggests that these are not viable tool inhibitors for in vivo studies (Koppaka et al., 2012). The exceptions include disulfiram and Win18446, which are broadly validated for in vivo use and have reported effects across tumor types (Kim et al., 2016; Rebollido-Rios et al., 2020).

Reversible ALDH1a inhibitors have become a rapidly developing area (Kundu and Iyer, 2023). A series of lactone/quinolone structures have been discovered for targeting various ALDH1 isoforms, with the majority of these studies showing IC50/Ki values in the 5–20 µM range and limited isoform selectivity (Buchman et al., 2015; Chefetz et al., 2019; Condello et al., 2015; Jiménez et al., 2019; Liang et al., 2020; Morgan and Hurley, 2015). Only four studies thus far have developed compounds in the 100–500 nM range, and these are more active against ALDH1a1 than other isoforms (Haenisch et al., 2021; Huddle et al., 2018; Yang et al., 2018) with the exception of CM121, a novel structure with IC50 values for ALDH1a2 and ALDH2 in the 500 nM range (Chen et al., 2018). Research in the contraception space has also led to the development of modestly potent ALDH1a1 and ALDH1a2 inhibitors; however, these contain a labile carbonyl that would react semicovalently at the active site (Chen et al., 2018; Kimble-Hill et al., 2014), making these undesirable leads. A review of all known inhibitors against the ALDH family as of 2017 exhaustively measured IC50 values and confirmed that no known inhibitor exhibited isoform selectivity nor worked in the <200 nM range (Yasgar et al., 2017). The last 10 years have witnessed an acceleration in new compound discovery, including compounds we developed with IC50 values of 5 nM in cellular assays (Son et al., 2023). As these programs move toward clinical development for immune and metabolic applications, they will provide validation for the potential of blocking retinoid signaling.

## Perspectives on retinoids in human health: Future directions

Few biological pathways have been studied as extensively as the retinoid pathway, especially in cancer trials. This has resulted in overwhelming evidence reflecting, at minimum, a lack of benefit for retinoid nuclear receptor agonists in solid tumors and, at worst, considerable evidence of harm in many patient settings. From early studies showing amplification of the retinoid synthetic enzymes in many aggressive cancers to more recent studies highlighting its role in immune tolerance, it should be apparent that retinoid nuclear receptor agonists do not have a role in cancer treatment outside of specific hematologic and cutaneous settings. The clinical profile of synthetic RAR/RXR agonists further highlights the key adverse cardiometabolic and bone effects that follow ectopic activation of either pathway. At the very least, these data suggest that only 13-cis retinoid species be considered for systemic testing rather than RAR activators, except in the very narrow disease areas where RAR/RXR agonists are known to exhibit overwhelming evidence of efficacy. Alternately, the rapid development of ALDH1 inhibitors and next-generation RAR antagonists offers promise in the context of precision medicine wherein patients or diseases with either amplification of specific ALDH1a enzymes or those where retinoid agonism accelerated disease should be prioritized for early clinical development.

In contrast to the often confusing results from studies of retinoid agonists, genetic studies have begun to unify our understanding of endogenous retinoid signaling in development, immunity, reproduction, and disease. These data suggest key opportunities for retinoid antagonists to be tested in certain cardiovascular diseases such as diabetes mellitus, obesity, and vascular disorders, various immunological diseases including irritable bowel syndrome or GVHD, as a male contraceptive, and in various cancers such as lung, prostate, gastric, sarcomas, and T-ALL. In the next decade, retinoid pathway antagonists currently entering clinical use will determine the potential for inhibiting the retinoid pathway.
